# Animal Model of Gestational Diabetes Mellitus with Pathophysiological Resemblance to the Human Condition Induced by Multiple Factors (Nutritional, Pharmacological, and Stress) in Rats

**DOI:** 10.1155/2016/9704607

**Published:** 2016-06-09

**Authors:** Siti Hajar Abdul Aziz, Cini Mathew John, Nur Intan Saidaah Mohamed Yusof, Massita Nordin, Rajesh Ramasamy, Aishah Adam, Fazlin Mohd Fauzi

**Affiliations:** ^1^Department of Pharmacology and Chemistry, Faculty of Pharmacy, Universiti Teknologi MARA, 42300 Bandar Puncak Alam, Selangor Darul Ehsan, Malaysia; ^2^Department of Physiology and Pharmacology, Faculty of Medicine, University of Calgary, 3330 Hospital Drive NW, Calgary, AB, Canada T2N 4N1; ^3^Department of Pharmaceutical and Life Sciences, Faculty of Pharmacy, Universiti Teknologi MARA, 42300 Bandar Puncak Alam, Selangor Darul Ehsan, Malaysia; ^4^Immunology Unit, Department of Pathology, Faculty of Medicine and Health Sciences, Universiti Putra Malaysia (UPM), 43400 Serdang, Selangor Darul Ehsan, Malaysia

## Abstract

This study attempts to develop an experimental gestational diabetes mellitus (GDM) animal model in female Sprague-Dawley rats. Rats were fed with high fat sucrose diet, impregnated, and induced with Streptozotocin and Nicotinamide on gestational day 0 (D0). Sleeping patterns of the rats were also manipulated to induce stress, a lifestyle factor that contributes to GDM. Rats were tested for glycemic parameters (glucose, C-peptide, and insulin), lipid profiles (total cholesterol, triglycerides, HDL, and LDL), genes affecting insulin signaling (IRS-2, AKT-1, and PCK-1), glucose transporters (GLUT-2 and GLUT-4), proinflammatory cytokines (IL-6, TNF-*α*), and antioxidants (SOD, CAT, and GPX) on D6 and D21. GDM rats showed possible insulin resistance as evidenced by high expression of proinflammatory cytokines, PCK-1 and CRP. Furthermore, low levels of IRS-2 and AKT-1 genes and downregulation of GLUT-4 from the initial to final phases indicate possible defect of insulin signaling. GDM rats also showed an impairment of antioxidant status and a hyperlipidemic state. Additionally, GDM rats exhibited significantly higher body weight and blood glucose and lower plasma insulin level and C-peptide than control. Based on the findings outlined, the current GDM animal model closely replicates the disease state in human and can serve as a reference for future investigations.

## 1. Introduction

Gestational diabetes mellitus (GDM), a common pregnancy complication, is defined by the American Diabetes Association as diabetes that is not clearly apparent diabetes, diagnosed in the second or third trimester of pregnancy [1]. During pregnancy, mothers undergo several metabolic changes to meet the energy demands of the fetus [2]. Resistance to insulin escalates to increase the glucose supply to the fetus. Pancreatic beta cells then compensate for the increased demand in glucose, and a normoglycemic state is maintained. However, women who develop GDM have deficits in beta cells response leading to insufficient insulin secretion, consequently leading to a state of hyperglycemia [2, 3]. This insulin resistance seen in GDM is similar to that observed in Type 2 diabetes mellitus (T2DM). When beta cells are no longer able to compensate for the insulin resistance, this then leads to glucose intolerance. A large percentage (over 25%) of women developed an abnormal glucose tolerance in pregnancy, but their glucose tolerance is most likely to return to normal postpartum [4, 5]. Decrease of insulin receptors on cell surfaces has also been associated with insulin resistance. The number of insulin receptors on monocytes has been found to be decreased in GDM [6]. Insulin receptor binding to monocytes increases in pregnancy and in midpregnancy but is significantly decreased in late pregnancy [6]. The insulin concentration necessary to reduce insulin binding by 50% (ID50) is lower in GDM diagnosed in late pregnancy [7]. Women diagnosed with diabetes during gestation have an increased incidence of complications during pregnancy as well as an increased risk of developing Type 2 diabetes mellitus (T2DM) later in life [8]. Additionally, offspring born to GDM mothers have an increased incidence of perinatal complications and an increased risk of obesity and T2DM later in life [9].

Observations of tissues and organs from pregnant women with and without GDM would expand our understanding of the disease. Yet, the scarcity of the samples and the lack of modalities make understanding the molecular mechanism and finding possible therapeutics for GDM difficult. For these reasons, animal models deliver an attractive alternative in studying the molecular mechanisms and treatment options for GDM. Initially, GDM animal models were induced solely by injecting diabetogenic agents such as Streptozotocin (STZ) and alloxan in low and high dosages [10–12]. However, it has been reported that those substances cause a complete or partial ablation of pancreatic beta cells and insulin deficiency instead of the consequences of insulin resistance [13]. In addition, the appropriateness of this model for GDM has been questioned as mean glucose exceeded 350 mg/dL, and diabetes of such severity rarely occurs in humans [14]. Meanwhile, complete or partial ablation of pancreatic beta cells results in a low rate of fetal malformations, which is not desirable [15]. In T2DM animal model, Nicotinamide (NA) is injected just before STZ to protect the pancreas from damage [16]. The combination of STZ + NA was then adopted in GDM animal model [17]. Diet also plays an important role in developing GDM animal models as fat intake affects glucose intolerance and elicits insulin resistance [14, 18]. Rats fed with high fat diet develop obesity and hyperinsulinemia but do not cause frank or effective diabetes [19]. However, diet high in fat and sucrose induced rapid obesity-related metabolic syndrome [20–23]. Abdel-Reheim et al. [24] proposed the combination of minimal dose of STZ with high fat sucrose diet (HFSD) in developing GDM animal model and proved that this combination was successful. Srinivisan et al. [25] highlighted the combination of low dose STZ-treated rats and high fat diet in inducing insulin resistance. In our previous study [26], we used the combination of STZ, NA, and high fat sucrose diet in our GDM animal model and evaluated the effect on Tregs, proliferation of splenocytes, production of reactive oxygen species (ROS) by neutrophils, and serum glucose levels [27].

In addition to the combination of STZ, NA, and HFSD in our previous GDM animal model, we now include stress in the current model. GDM pregnancies are linked with a heightened level of oxidative stress, where sleeplessness or disturbance in sleep increases stressand consequently impairs antioxidant defense system [28]. Mothers' oxidative balance imposes a great impact on fetus development, in addition to the mother's health.

Hence, the current study investigates whether the combination of nutritional manipulation, pharmacological treatment, and stress induction can create appropriate immunometabolic changes in pregnant rat, which can be developed as a model for understanding the consequences of GDM as well as providing insights into potential treatments and preventative measures. To the authors' knowledge, GDM animal model induced with the previously mentioned factors has not been published in the scientific literature and hence recorded as a pilot study. Changes on glycemic and lipid parameters along with proinflammatory cytokines level and oxidative stress in the rats were observed in this study to evaluate the appropriateness of our animal model.

## 2. Materials and Methods

### 2.1. Experimental Animals

Female Sprague-Dawley rats aged 8-9 weeks were procured from the Laboratory Animal Facility and Management (LAFAM), UiTM Puncak Alam Campus, Malaysia. A total of 45 female rats were randomly taken for the study, where 10 rats were assigned as control and the rest were assigned as GDM group. Animals were maintained in an experimental room under the following conditions: (a) temperature of 22 ± 2°C, (b) humidity of 50 ± 10%, and (c) illumination of a 12-hour light/dark cycle for control and 18-hour light/4-hour dark cycle for GDM group. Changes in sleeping cycle and environmental status have been shown to induce abnormally high levels of oxidant stress [29]. All experimental procedures presented in this study were approved by the Research Ethics Committee (Ethics Number: 133/2015) of Universiti Teknologi MARA (UiTM).

### 2.2. Dietary Intake

Standard diet (Gold Coin, Malaysia) was given to control rats. Meanwhile, GDM group rats were fed with HFSD (SP-11032, Australia) from Week 10 onwards. HFSD consist of 25% sucrose, 40% beef tallow, and 20% of casein protein.

### 2.3. Smearing and Breeding of Animals

**Figure 1:**
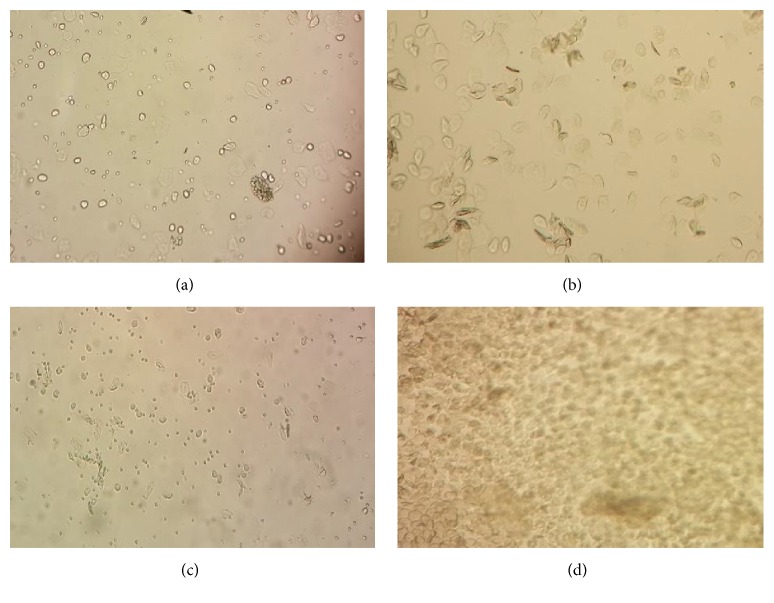
Photomicrograph of the vaginal smears of rat showing estrous cycle stages. (a) Proestrous stage (round nucleated epithelial cells); (b) estrous stage (cornified or irregular shape of epithelial cells); (c) metaestrous stage (low number of round cells); and (d) diestrous stage with mostly small and round cells.

**Figure 2:**
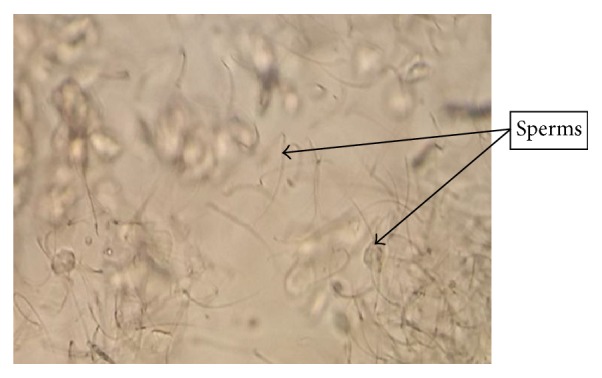
Photomicrograph of the vaginal smears of rat showing the existence of sperms. Sperms were visible and observed during vaginal smears after successful mating.

After one week of acclimatization, animals were examined for estrous cycles for 2 consecutive weeks. Rats follow a 4-day pattern of estrous cycle, namely, estrous (E), metaestrous (M), diestrous (D), and proestrous (P). Rats in estrous stage (Figure 1(b)) were allowed to mate overnight with resident males from the same strain at a source ratio of 2 males per 1 female. Pregnancy was confirmed through vaginal smear as shown in Figure 2, whereby copulation was confirmed by detection of sperm. All tests were initiated when pregnancy was confirmed, denoted as D0.

### 2.4. Pipette Smear Technique

In this technique, vaginal secretion from each rat was collected using a plastic pipette containing ~1-2 mL of normal saline (NaCl 0.9%) every morning. Vaginal secretion was collected by inserting the pipette tip into the rat's vagina and flushing the cells from the vaginal lining. One or two drops of vaginal secretion were placed onto a clean glass slide. Separate glass slides were used for each cage of animals. Unstained vaginal secretions were directly viewed under a light microscope at 40x magnification. Three types of cells were observed, which were round and nucleated epithelial cells, nonnucleated irregular cornified cells, and small round cells (Figure 1). These characteristics of cells were used for the determination of estrous cycle phases [30]. Rats that do not follow a 4-day pattern of estrous cycle were excluded from the study.

### 2.5. Induction of Experimental GDM

Streptozotocin (STZ) (Sigma Aldrich, USA) was induced on D0 by a single intraperitoneal (ip) injection at a dose of 35 mg/kg bw in 0.1 mol/L citrate buffer (pH 4.5). Nicotinamide (NA) (Sigma Aldrich, USA) 120 mg/kg bw was induced ip 15 minutes prior to STZ. Control rats received an equal volume of citrate buffer only. The rats were returned to their respective cages and blood glucose levels were analysed 72 hours after STZ administration. Rats with stable hyperglycemia were selected for further study where on D6 half of the animals were euthanized after initial results were collected and the rest were sacrificed after the final results were collected.

### 2.6. Blood Samples

Blood samples were collected through cardiac puncture for biochemical and hematological analysis on D6 and D21. Serum was collected into plain tubes while plasma was collected into heparin tubes. Collected blood in plain tube was left to clot for half an hour. Subsequently, heparin tubes were kept cold in an ice box to prevent clotting. Both tubes were centrifuged at 2500 rpm for 12 min at −4°C. Serum and plasma were collected in microcentrifuge tubes and kept at −80°C for further analysis.

### 2.7. Food Intake and Body Weight Changes

Food intake was calculated daily by calculating the difference between the amount of food given and the amount of residual food at the end of each day.

### 2.8. Laparohysterectomy

Six animals were sacrificed on D6 to acquire the initial results, and the rest was sacrificed on D21. Rats were anesthetized with diethyl ether and thoracic, abdominal, and pelvic regions were dissected. Abnormalities in the internal organs were examined. Uterus and ovaries were excised and exposed. The number of corpora lutea on each ovary was identified and total gravid weight was noted prior to opening the uterus. To find out the implantation loss, uterus was kept in ammonium sulphide (10%) solution for 5 minutes [31]. The weight, number, sex, and location of fetus and implantation sites along with placenta were recorded.

### 2.9. Measurement of Glucose Level

Tail incision method was used to measure weekly fasting glucose level using hemoglucometer (Lifescan, Johnson and Johnson, USA) and glycemic levels were monitored throughout the experiment. Serum glucose was measured upon sacrifice using commercially available reagent kits (ILab Chemistry Analyzer 300 PLUS; Instrumentation Laboratory, USA).

### 2.10. Measurement of Insulin Level

Insulin level was assayed using Mercodia Rat Insulin ELISA kit (Sweden). The optical densities of the samples were read at 450 nm. The concentration of insulin level was obtained by computerized data reduction of the absorbance for the calibrators, except for calibrator 0, versus the concentration using cubic spline regression.

### 2.11. Measurement of C-Peptide

Plasma C-peptide level was assayed by Mercodia Rat C-peptide ELISA kit (Sweden). The optical densities of the samples were read at 450 nm. The concentration of C-peptide was obtained by using cubic spline regression the same way as the Insulin ELISA kit mentioned above.

### 2.12. Measurement of C-Reactive Protein

Blood plasma was used for the determination of C-reactive protein, using full range CRP (frCRP) commercial kit purchased from Randox (All Eight (M) Sdn. Bhd). Liquid assayed specific protein control levels 1 and 2 were used as control. After preparation of blood samples, 500 *μ*L plasma was transferred to each Selectra tube, and the results were obtained using a Vita lab Selectra machine.

### 2.13. Biochemical Measurements

Serum levels of triglycerides, total cholesterol, LDL-cholesterol, and HDL-cholesterol were measured using ILab Chemistry Analyzer 300 PLUS (Instrumentation Laboratory, USA).

### 2.14. Measurement of Gene Expression

Organs for gene expression studies were harvested upon sacrifice and were immediately stored in liquid nitrogen for further investigations. Total RNA of tissues of glucose related genes (GLUT-2, GLUT-4, AKT-1, IRS-2, and PCK-1) and antioxidant genes (SOD, CAT, and GPX) was isolated from the liver while tissues of inflammatory genes (IL-6 and TNF-*α*) were isolated from spleens RNase Mini Kit (Qiagen, USA) according to the manufacturer's instructions. Purity of the extracted RNA was determined by measuring the ratio of the optical density at 260 and 280 nm using a spectrophotometer (BioRad, USA), which ranged between 1.8 and 2.0. mRNA expressions of the genes were determined by qPCR as described in our previous study, John et al. [26]. Primers specific for respective genes and beta-actin genes (housekeeping gene) were designed from the gene sequence of rat (*Rattus norvegicus*) adapted from NCBI (National Center for Biotechnology Information) GenBank Database [32]. The oligonucleotide sequences of primers used for qPCR, QuantiTect Primer Assay, were purchased from Qiagen USA. Accession numbers of each gene are IRS-1 (NM_012969), Akt-1 (NM_033230), Slc2a2_1 (NM_012879), Rn_Slc2a4_1 (NM_012751), TNF-*α*-_1 (NM_012675), IL-6 (NM_012589), GPX (NC_005107.4), SOD (NM_017050.1), CAT (NM_012520.1), and ACTB (NM_031144.2) as housekeeping gene. The expression for each sample was measured according to the quantity of *β*-actin expressed, while the number of fold expressions was calculated using 2^−ΔΔCt^.

### 2.15. Histology Study

Histology of the pancreas was done to observe the morphological changes. Pancreas tissues were extracted from the rats upon sacrifice on D21 from each group. Pancreas harvested was stored in 10% formalin solution before proceeding with histology study. Haematoxylin and Eosin staining procedure was employed in this experiment.

### 2.16. Statistical Analysis

GraphPad Prism software was used for statistical analysis using *t*-test, which compares the different parameters between control and GDM group. Value *P* < 0.05 was considered significant. Data collected for glycemic and lipid parameters were subjected to Hierarchical Clustering and Principal Component Analysis [33] to visualize the variation between control and GDM group. However, parameters which involve gene expressions could not be performed using hierarchical clustering and PCA as control is set and normalized to 1. Hence, this could give a misleading result. Both hierarchical clustering and PCA were performed in R. 

## 3. Results

### 3.1. Glycemic Parameters (Glucose, Insulin, and C-Peptide) and Body Weight

**Figure 3:**
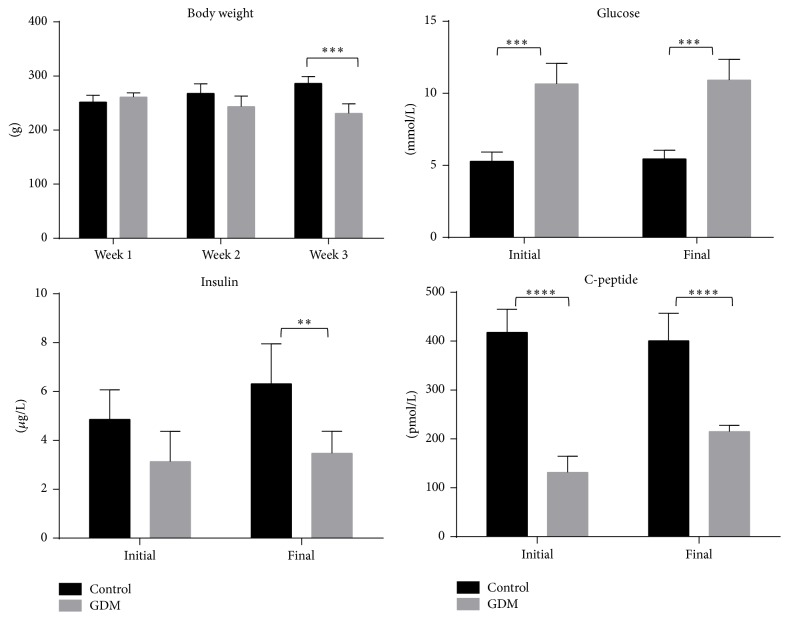
Body weight, glucose level, insulin level, and C-peptide of both control and GDM group. Body weight of GDM-induced rat was decreased from Week 1 to Week 3 compared to control rat, where the body weight was increased significantly. Glucose level was higher in GDM group compared to control. Levels of insulin and C-peptide were higher in control compared to GDM group. Data shown as mean ± SD of four rats. For each parameter, a value with the asterisk signifies *P* < 0.05 and the absence of asterisk indicates otherwise. *P* value less than 0.01 was designated with two (*∗∗*) asterisks, *P* value less than 0.001 was designated with three (*∗∗∗*) asterisks, and *P* value less than 0.0001 was designated with four (*∗∗∗∗*) asterisks.

**Figure 4:**
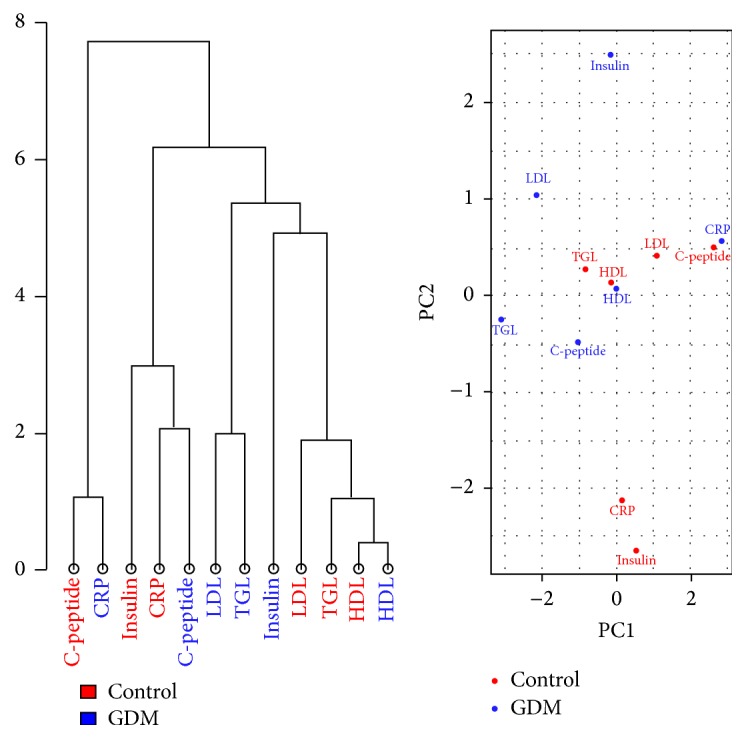
Hierarchical Clustering and Principal Component Analysis for both glycemic and lipid parameters. Both graphs showa clear separation between the glycemic and lipid parameters in both GDM group and control. However, there is an exception to this in the case of HDL.

Weekly fasting serum glucose levels are shown in Figure 3. Glucose level in GDM groups was significantly higher (*P* < 0.05) than control, confirming the hyperglycemic state at both initial (D6) and final (D22) phases of pregnancy. Additionally, insulin and C-peptide levels of GDM group showed a significant increase from the initial phase to the final phase of pregnancy, indicating increase of insulin production in the final phase of pregnancy. A slight decrease in body weight was observed from Week 1 to Week 2 and from Week 2 to Week 3 of GDM group as shown in Figure 3. Rats in control group experienced a slow increase of body weight. From both hierarchical clustering and PCA (see Figure 4), it can be seen that there is a clear separation between control and GDM for all glycemic parameters. There were no notable changes in food intakes for both control and GDM groups (data not shown).

### 3.2. The Lipid Parameters

**Figure 5:**
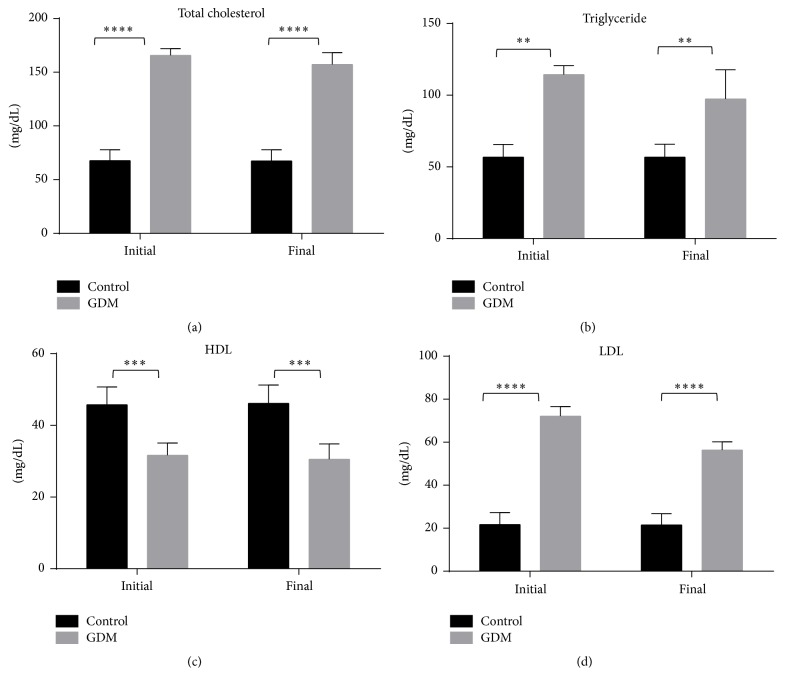
Lipid profile of both control and GDM group. Levels of total cholesterol, triglyceride, and LDL were higher in GDM group compared to control while level of HDL was higher in control compared to GDM group. Data shown as mean ± SD of four rats. For each parameter, a value with the asterisk signifies *P* < 0.05 and the absence of asterisk indicates otherwise. *P* value less than 0.01 was designated with two (*∗∗*) asterisks, *P* value less than 0.001 was designated with three (*∗∗∗*) asterisks, and *P* value less than 0.0001 was designated with four (*∗∗∗∗*) asterisks.

The data in Figure 5 shows the changes of lipid profile of both control and GDM groups at the initial and final phases of pregnancy. It can be seen from Figure 4 that there was an increase in total cholesterol, triglyceride, and LDL-cholesterol (*P* < 0.001) from the initial to the final phases of pregnancy in the GDM group compared to the control group. Additionally, HDL-cholesterol was significantly decreased in the GDM group compared to the control group. From both hierarchical clustering and PCA (see Figure 4), it can be seen that there is a clear separation between control and GDM for all lipid parameters, with the exception of HDL.

### 3.3. Plasma CRP and Proinflammatory Genes

**Figure 6:**
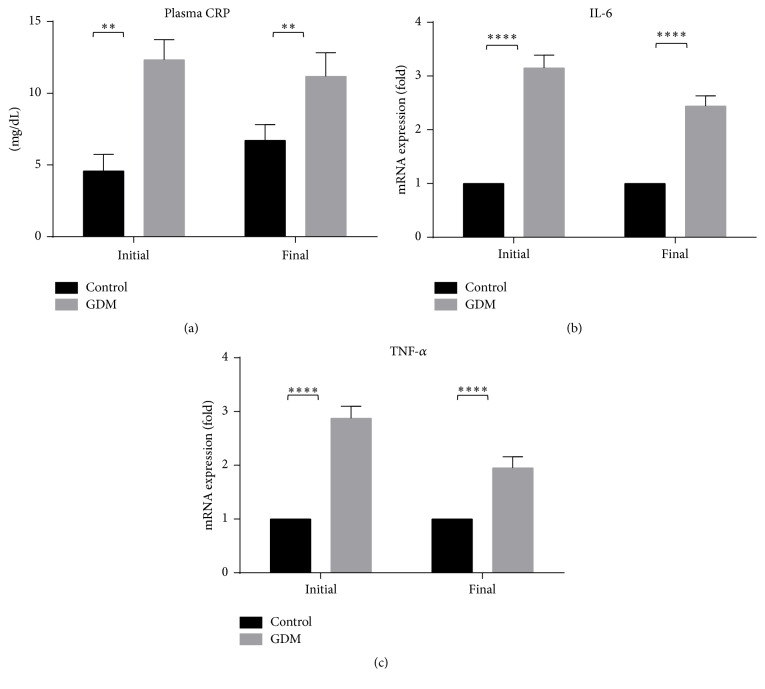
Plasma CRP and expression of inflammatory genes of control and GDM rats. Level of plasma CRP at initial and final stages was high in GDM group compared to control group. mRNA expressions of IL-6 and TNF-*α* are shown. mRNA levels of inflammatory genes show that both IL-6 and TNF-*α* were higher in GDM group compared to control. Data shown as mean ± SD of four rats. For each parameter, a value with the asterisk signifies *P* < 0.05 and the absence of asterisk indicates otherwise. *P* value less than 0.01 was designated with two (*∗∗*) asterisks, and *P* value less than 0.0001 was designated with four (*∗∗∗∗*) asterisks.

Figure 5 shows the level of plasma CRP and both inflammatory genes (IL-6 and TNF-*α*) at the initial and final phases of pregnancy in GDM and control groups. The level of plasma CRP is higher in GDM group compared to control group at both phases of pregnancy which stimulates the acute phase inflammatory response (see Figure 6(a)). As shown in Figures 6(b) and 6(c), both IL-6 and TNF-*α* levels increase (*P* < 0.001) from the initial and final phases of pregnancy in GDM group, but no significant changes were observed in the control group. In addition, in both phases, the level of IL-6 and TNF-*α* in GDM groups was higher than control.

### 3.4. Expression of Genes Related to Glucose Metabolism

**Figure 7:**
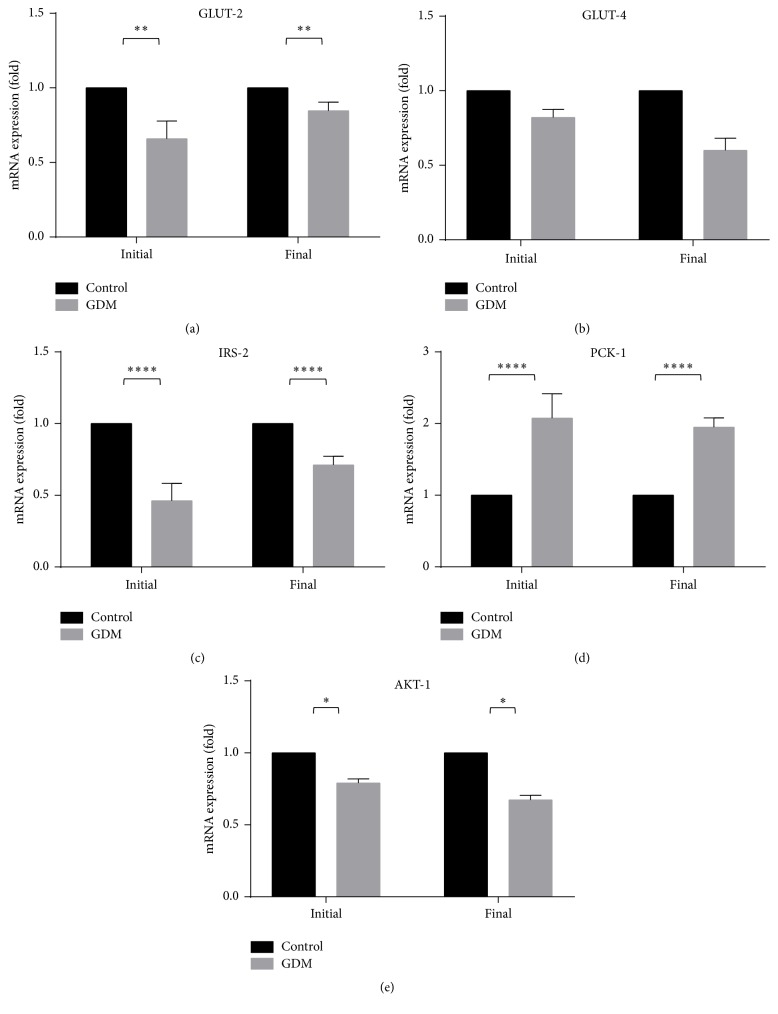
Expression of glucose related genes of control and GDM rats. GLUT-2, GLUT-4, IRS-2, and AKT-1 levels were lower in the GDM group than control. The opposite was true for PCK-1. Data shown as mean ± SD of four rats. For each parameter, a value with the asterisk signifies *P* < 0.05 and the absence of asterisk indicates otherwise. *P* value less than 0.05 was designated with one (*∗*) asterisk, *P* value less than 0.01 was designated with two (*∗∗*) asterisks, and *P* value less than 0.0001 was designated with four (*∗∗∗∗*) asterisks.

Genes involved in glucose transporter (GLUT-2 and GLUT-4) and insulin signaling pathway (IRS-2, PCK-1, and AKT-1) at the initial and final phases of pregnancy in the control and GDM group are shown in Figures 7(a)–7(e). GLUT-2, GLUT-4, AKT-1, and IRS-2 levels were lower in the GDM group than control.

### 3.5. Expression of Antioxidant Genes

**Figure 8:**
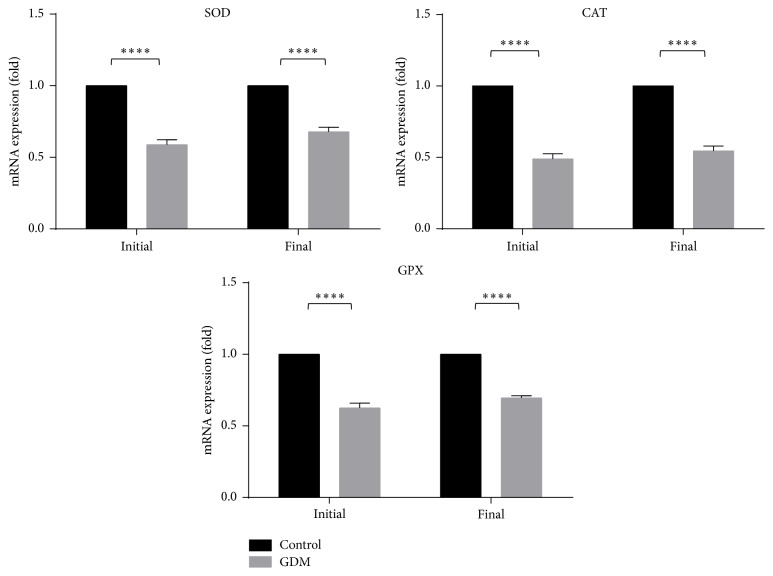
Expression of antioxidant genes of control and GDM rats. SOD, CAT, and GPX genes were lower in the GDM group compared to control, in both initial and final phases at final phase of GDM compared to the initial phase. Data shown as mean ± SD of four rats. For each parameter, a value with the asterisk signifies *P* < 0.05 and the absence of asterisk indicates otherwise. *P* value less than 0.0001 was designated with four (*∗∗∗∗*) asterisks.

The expressions of antioxidant genes for SOD, CAT, and GPX were shown in Figure 8. All the genes were lower (*P* < 0.001) in the GDM group compared to control, in both initial and final phases.

### 3.6. Histology of Pancreas

**Figure 9:**
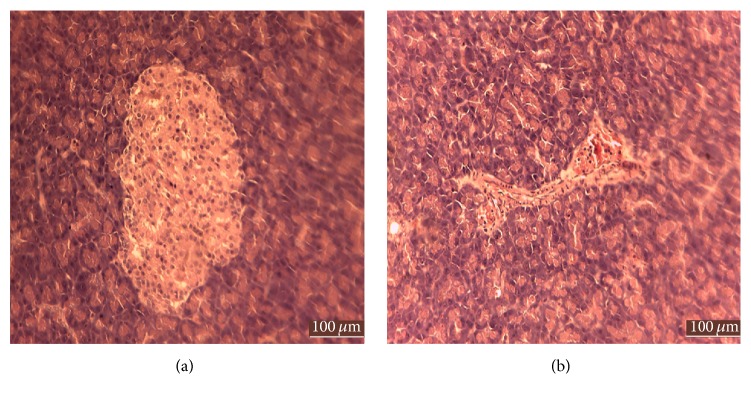
Histology of pancreas of control and GDM group. (a) H & E staining on normal pancreas showing undestroyed endocrine and exocrine gland. (b) H & E staining on STZ-treated pancreas showing slightly destroyed Islet of Langerhans.

Histology section of pancreas was shown in Figure 9. Pancreas of control group shows an unaffected structure of endocrine gland while pancreas of GDM group shows deteriorated endocrine glands, which confirmed the protective effect of NA on the damage to beta cells within the Islet of Langerhans caused by STZ.

### 3.7. The Maternal Reproductive Status

Table 1 shows the maternal reproductive status of both control and GDM groups. The average number of live fetuses, number of dead fetuses, gravid weight, empty weight, number of implantations, and sex ratio were significantly lower in the GDM group compared to control. The lower average number of live fetuses in the GDM group confirmed the restriction in the intrauterine growth. GDM groups showed a higher number of postimplantation loss sites, preimplantation loss, placental weight, fetal weight, fetal length, and number of corpora lutea than control. The fetal weight of the GDM group was higher than control, which confirms the macrosomia (large for gestational age) status of the fetuses.

## 4. Discussion

Current animal model combines multiple factors that cause insulin resistance in gestation. This study provides data associated with glycemic parameters, lipid parameters, glucose transporters parameters, genes affecting insulin signaling, proinflammatory cytokines parameters, and oxidative parameters on the initial and final phases of pregnancy in both control and GDM groups. In our study, body weight of rats on first week in the GDM group was higher compared to the control group, which can be attributed to consumption of high fat sucrose diet [34]. A decrease in body weight of rats was observed on the following weeks in the GDM group (Figure 3), despite no notable changes in food intake. It should be noted that a decrease in body weight is not usually seen in human GDM. This decrease in body weight of GDM rats may be due to insulin resistance induced by STZ, due to the partial damage inflicted on the pancreatic beta cells [35]. This then promotes the catabolism of fats and proteins, resulting in weight loss [36]. This decreasing trend of the weight of GDM rats was also seen in the study by Abdel-Reheim et al., [24] where the same dose of STZ (35 mg/kg) was also used in this study. It was noted by Abdel-Reheim et al. [24] that this trend was not seen when a dose of 20 mg/kg STZ was used. Meanwhile, average glucose level was higher in GDM group compared to control group, confirming the glycemic state of rats. Insulin level was lower in GDM group compared to the control group, which is also supported by the low level of C-peptide (measuring insulin production). These results suggest that rats with GDM experienced a higher rate of insulin resistance compared to the control rats, which leads to decrease in insulin secretion. If beta cells are not able to compensate for the increase in insulin resistance, this can lead to GDM [37]. It can be noted that insulin and C-peptide levels were significantly lower than usual (>300 pmol/L) [37]. One possible reason for this is the additional stress factor induced in this animal model. Stress has been linked to low insulin level due to the production of free radicals. Further explanation of this can be found in the discussion of antioxidant levels of GDM rats later.

A state of hyperlipidemia is commonly observed in GDM [38]. This was also observed in the GDM groups where level of total cholesterol, triglyceride, and LDL were increased from the initial to the final phases whereas level of HDL was decreased (Figure 4). Increase in triglycerides level may be due to the absorption of fat from small intestine due to HFSD intake, where fatty food leads to increase in visceral fat deposition in the early stage of pregnancy. These events can lead to GDM [39]. In addition, HFSD feeding increases plasma free fatty acid (FFA) concentrations and causes insulin resistance by inhibiting insulin-stimulated glucose uptake, glycogen synthesis, and/or phosphorylation activity [40]. In line with this, a downregulation of GLUT-4 was also observed in this study, indicating low glucose translocation and hence low glucose uptake in the skeletal muscles.

In the evaluation of proinflammatory cytokines, our results show that TNF-*α* level and IL-6 levels are higher in the GDM group compared to control (see Figure 5). Obesity and T2DM, which are associated with insulin resistance, have been shown to have fat cell dysfunction that results in the production of an excessive amount of proinflammatory adipokines such as IL-6 and TNF-*α* [41]. This excessive production of IL-6 and TNF-*α* may also be a result of oxidative stress and inflammatory changes caused by hyperglycemia [42, 43]. TNF-*α* is believed to induce insulin resistance by a number of mechanisms such as increase in serine phosphorylation of IRS-1, which disrupts the insulin signaling cascade [44]. In addition, several studies have shown that there is an inverse correlation between IL-6 concentration and insulin response [45], although the mechanism behind it has not been elucidated. In line with the higher level of IL-6 and TNF-*α*, serum CRP level (inflammatory marker) was also higher in GDM than control (see Figure 5(a)). Elevated levels of plasma CRP have been reported to enhance the development of diabetes [46] by interacting with cytokines (IL-6 and TNF-*α*) in stimulating the acute phase inflammatory response [47, 48]. The higher level of proinflammatory cytokines and plasma CRP thus indicates the likelihood of insulin resistance in our GDM model.

In the evaluation of genes affecting insulin signaling pathway, GLUT-2, GLUT-4, AKT-1, and IRS-2 levels were lower in the GDM group than control. The opposite was true for PCK-1. In GDM, fatty-sucrose diet promotes serine phosphorylation of IRS-1, reducing its ability to act as an insulin receptor substrate [49–52], thereby reducing GLUT-4 translocation to the plasma membrane. Serine phosphorylation of IRS also deactivates AKT signaling cascade, which inhibits glycogen synthesis, suppressing the gluconeogenesis in liver of GDM rat [53]. AKT have a secondary role in the production of glycerol which releases FFA into the blood stream [54]. As mentioned previously, elevated FFA is associated with insulin resistance. The lower concentration of IRS-2 genes and AKT-1 in GDM group and reduced level of GLUT-4 from the initial to final phases indicate the possible defect of insulin signaling in our GDM model. Furthermore, the higher level of PCK-1 in the GDM model compared to control indicates that lipid metabolism and glucose homeostasis are compromised, leading to insulin resistance [55].

Antioxidant levels, SOD, GPX, and CAT (Figure 7), were evaluated in this study to analyze the effect of stress on GDM rats. It can be seen that all antioxidant genes were significantly lower in the GDM group than control, indicating that GDM rats were under stress. This result is also in line with the findings of Sindhu et al., [41] where diabetic rats showed reduced activity of the same antioxidant enzymes. Ornoy [56] also observed a significant decrease in endogenous antioxidant enzymes when oxidative stress was induced on embryos under diabetic condition. Both studies also highlight that impaired antioxidant status can be linked to oxidative stress associated with diabetes stress due to an overproduction of reactive oxygen species (ROS) [57]. ROS modulates the insulin signaling pathway* via* two mechanisms. The first mechanism involves the production of ROS in response to insulin where ROS is involved in physiological functions such as vascular homeostasis [58]. In the second mechanism, ROS negatively regulates the insulin pathway, consequently leading to reduced insulin secretion and consequently insulin resistance [59–61]. Hence, this underlines the link between stress and the pathophysiology of GDM.

Pancreas of the control group was undestroyed whereas in the GDM group, there was a slight destruction of the Islet of Langerhans (Figure 8). Effect of GDM on pancreas can be observed clearly after the process of staining. The partially destroyed Islets of Langerhans show the cytotoxic effect of STZ [62]. STZ causes the alkylation of DNA via GLUT-2 which later induces the activation of poly ADP-ribosylation (PARP), leading to depletion of cellular NAD+ and ATP [35]. Necrotic death of beta cells was partially prevented by action of NA in lowering the PARP activity, thus ensuring enough NAD and ATP to be used [63].

Maternal reproductive status (Table 1) was also evaluated in our study where one of the statuses observed was the weight of the fetus. It can be seen from Table 1 that GDM group showed a higher fetal weight than control. Macrosomia, or large gestational size, is caused by hyperinsulinemia to which insulin is one of the main growth factors during fetal life [64]. In addition, hyperglycemia of intrauterine milieu of GDM mother may cause the fetal endocrine pancreas to promote hyperinsulinemia. As previously shown, GDM rats showed a higher insulin and average glucose level than control. Another status observed was the number of live fetuses where GDM group had a lower number than the control. In a retrospective analysis done by Gunther et al., [65] intrauterine fetal death was more prevalent in pregnant women with diabetes (preconceptional diabetes mellitus and GDM) than women without diabetes. Additionally, the number of postimplantation loss sites was higher in GDM group which indicates that rats with GDM had newborns with intrauterine growth restriction.

## 5. Conclusion

In this study, we administered STZ and NA to pregnant rats, along with feeding them with HFSD and altering sleep patterns to induce GDM. The GDM animal model showed signs of insulin resistance where expressions of both proinflammatory cytokines (IL-6 and TNF-*α*), PCK-1, and serum CRP level were higher than control. Furthermore, low concentration of IRS-2 genes and AKT-1 and reduced level of GLUT-4 from the initial to final phases indicate the possible defect of insulin signaling in our GDM animal model. The impaired antioxidant status in our GDM model showed that inducing stress through changing sleeping cycle could induce oxidative stress, which is associated with diabetes. Our GDM animal model showed a higher body weight than control during Week 1, which was due to HFSD feeding. The GDM animal then showed a decrease in body weight from Week 1 to Week 3, due to the destruction of beta cells by STZ. Blood glucose level was also higher in GDM group than control, indicating the hyperglycemic state of the GDM rats. Higher level of lipid parameters (triglycerides, total cholesterol, and LDL-cholesterol) in GDM group then confirms the state of hyperlipidemia in GDM rats. Based on these results, it can be concluded that suitable GDM animal model can be created through nutritional, pharmacological, and lifestyle manipulations. This model can then be used to further understand the pathophysiology of GDM and consequently finding novel therapies for GDM.

## Figures and Tables

**Table 1:** Data shown below is the reproductive performances of control group and GDM group. Data shown as mean ± SD of four rats. For each parameter, a value with the asterisk signifies *P* < 0.05 and the absence of asterisk indicates otherwise. *P* value less than 0.05 was designated with one (*∗*) asterisk, *P* value less than 0.01 was designated with two (*∗∗*) asterisks, *P* value less than 0.001 was designated with three (*∗∗∗*) asterisks, *P* value less than 0.0001 was designated with four (*∗∗∗∗*) asterisks, and *P* value less than 0.00001 was designated with five (*∗∗∗∗∗*) asterisks.

Group	Control	GDM
Live fetus	8.57 ± 0.23	5.23 ± 0.12^*∗∗∗∗*^
Number of dead fetuses	0.32 ± 0.18	0.22 ± 0.13
Gravid wt.	67.23 ± 1.30	65 ± 1.73
Empty uterus weight	6.23 ± 0.12	4.32 ± 0.02^*∗∗∗∗∗*^
Placental wt.	4.02 ± 0.13	4.82 ± 0.04^*∗∗∗∗*^
Fetal wt.	3.08 ± 0.11	3.42 ± 0.11^*∗∗*^
Fetal length	2.89 ± 0.11	4.85 ± 0.11^*∗∗∗∗*^
Number of corpora lutea	11 ± 0.32	11.23 ± 0.21
Number of implantations	10 ± 0.23	6.56 ± 0.8^*∗∗∗*^
Pre IMP loss%	4.20 ± 1.32	7.51 ± 0.8^*∗∗*^
Post IMP loss%	1.22 ± 0.83	3.16 ± 1.12^*∗*^
Sex ratio (M/F)	1 ± 0.05	0.7 ± 0.02^*∗∗∗∗*^
